# Family Interviews Improve Health Service Recommendations in Mortality Review Process: A Mixed‐Methods Assessment

**DOI:** 10.1111/hex.70233

**Published:** 2025-04-09

**Authors:** Catherine Kothari, Fernando Ospina, Nia Evans, Cynthia Bane, Joi Presberry Dixon, Vaishali Patil, Ruth Butters, Rosemary Fournier, Susanna C. Joy, Brenda O'Rourke, Josephine Woods, Debra Lenz, Aaron L. Davies

**Affiliations:** ^1^ Department of Biomedical Sciences Western Michigan University Homer Stryker MD School of Medicine Kalamazoo Michigan USA; ^2^ National Center for Fatality Review and Prevention Okemos Michigan USA; ^3^ Kalamazoo County Health and Community Services Kalamazoo Michigan USA; ^4^ NAACP Kalamazoo Branch Kalamazoo Michigan USA; ^5^ Bronson Medical Group Kalamazoo Michigan USA

**Keywords:** community and patient engagement, health services improvement, population health

## Abstract

**Background:**

Within the United States, although there is strong motivation for incorporating family interviews into Fetal Infant Mortality Reviews (FIMR) and important potential for expanding it to other types of reviews, there is limited evidence that family interviews make a difference in review team outcomes.

**Objective:**

This study aims to assess the impact of FIMR family interviews identifying health service gaps and generating actionable improvements.

**Design:**

Mixed methods design with quantitative case‐control analysis and qualitative semi‐structured interviews. Quantitative data collection was secondary analysis of FIMR administrative records, comparing outcomes with and without family interviews using Generalized Estimating Equation (GEE) and descriptive analysis of annual FIMR team evaluation responses. Qualitative data collection included audio‐taping, transcribing and consensus coding of semi‐structured interviews.

**Setting and Participants:**

The quantitative setting was Kalamazoo County, Michigan, FIMR reviews from 2015 to 2023, whereas the qualitative setting was virtual. Quantitative sample was the full population of completed FIMR case reviews (*N* = 158), and the 15 FIMR team case reviewers completing the 2023 annual evaluation. The qualitative sample was a purposive sample of 28 FIMR administrators across the United States.

**Main Outcome Measures:**

Quantitative outcomes were review‐identified contributing stressors and subsequent recommendations generated in the case review process. Qualitative outcomes were thematic experiences of family interview implementation and impact within mortality review process.

**Results:**

The 53 cases (34%) with family interviews were similar to the 105 (66%) non‐interview cases regarding multipleperinatal characteristics and were different regarding death type and manner. Controlling for these differences, GEE analysis found that family interviews were associated with increased identification of stressors, especially medical and socioeconomic, with a 2.6 increase in the number of stressors identified (aOR = 2.6, 95% CI: 1.5–4.7, *p* < 0.001). Family interviews were associated with a 40% increase in recommendations generated (aOR = 1.4, 95% CI: 1.0–2.0, *p* = 0.05), especially regarding patient‐provider communication. Two‐thirds of the FIMR team reported that family interviews were ‘Very Impactful’ in making meaningful system changes. Qualitatively, three primary themes emerged: Hurdles to Getting Interviews, Completing the Picture and Bringing the Human Connection.

**Conclusions:**

This study drew upon multiple types of data, documenting the challenges obtaining interviews, while emphasizing their value identifying root causes, producing actionable healthcare service recommendations and motivating action.

**Patient or Public Contribution:**

Locally, members of community advocate organizations representing birthing and parenting individuals and those representing people identifying as Black race contributed by actively refining the implementation of FIMR family interviews, including how interview information was utilized in FIMR case review meetings (study interventions). Mock FIMR case review meetings conducted as part of United Way public ‘bus tours’ and with undergraduate/graduate university students further refined recommendations coding and dissemination strategies. Leadership from public and private health service organizations shared the facilitators and barriers for applying FIMR recommendations to institutional policy and practice (shaping the development of recommendations coding as well as dissemination and interpretation of study findings). Nationally, a network of mortality review funders and administrators, in a 4‐year learning series convened by the National Center for Fatality Prevention and Review, provided input in centreing family interviews within different types of mortality review processes (influencing study objectives, qualitative interview structure and results interpretation). Additionally, US policy leaders in maternal child health and health equity provided historical and current context for FIMR family interview funding and enabling legislation (informing the interpretation of study results and specifically referenced within the manuscript).

**Abbreviations**
CDR: Child death review
FIMR: Fetal infant mortality review
HRSA: Health resources and services administration
MCHB: Maternal child health bureau
MMR: Maternal mortality review
MPHI: Michigan public health institute
NFR‐CRS: National fatality review case reporting system

## 1 Introduction

Mortality review of sentinel events have long been important tools for identifying gaps in systems of care and informing prevention strategies [1, 2, 3]. The US Fetal Infant Mortality Review (FIMR) is a community‐based, action‐oriented process for reviewing deidentified fetal and infant deaths to identify potential contributors, develop recommendations and activate systems' improvements for women, infants and families [4]. FIMR's ability to achieve these outcomes relies upon effective implementation of each step in the process:

1. Assembling case information (records and family interview)
2. Conducting case review (multidisciplinary team of frontline providers and community advocates)
3. Disseminating, prioritizing and implementing recommendations

Among the plethora of morbidity and mortality reviews, present in nearly every healthcare institution across the United States, family interviews are unique to FIMR, although Maternal Mortality Review guidance documents are beginning to call for ‘informant interviews’ to better understand contextual contributors [5, 6]. Globally, parental engagement in perinatal mortality reviews is more common, and viewed as the key to important lessons in healthcare services improvement [7, 8]. An important example of this is the UK Perinatal Mortality Review Tool, which prioritizes supporting families in their grief process (96% successful outreach to parents) and eliciting family input for reviews (55% of parents provide feedback) [9]. Within FIMR, this foundational element is grounded in FIMR's explicit health equity focus, and is designed to provide insight into sociocultural circumstances and interpersonal experiences that are otherwise unknowable, particularly when they occur to populations that have been systematically disenfranchised, such as Black families and poor families [4]. FIMR family interviews elicit parents' and caregivers' perspectives, including the ways in which they were able, or not, to access services, how they were treated by service providers, the circumstances surrounding the life of their child, and the sequence of events leading to death [5, 10, 11]. Yet, teams report facing multiple barriers securing family interviews, primarily missing or inaccurate contact information, but also lack of resources (funding or staff), staff discomfort conducting family interviews and inadequate training [12]. Regardless, most FIMR teams (60%, 97 of the 162 in the United States) conduct family interviews; even if teams were only able to complete them for a minority (15%) of their cases [12]. In sum, although there is strong motivation for incorporating family interviews in the United States and important potential for expanding it to other types of reviews, there is limited evidence that family interviews make a difference in review team outcomes. Our overall study goal was to assess the impact of FIMR family interviews upon outcomes across the FIMR review process: identifying health system gaps, generating service recommendations and implementing recommended improvements.

## 2 Materials and Methods

### 2.1 Parallel Convergent Mixed‐Methods Design

**Figure 1:**
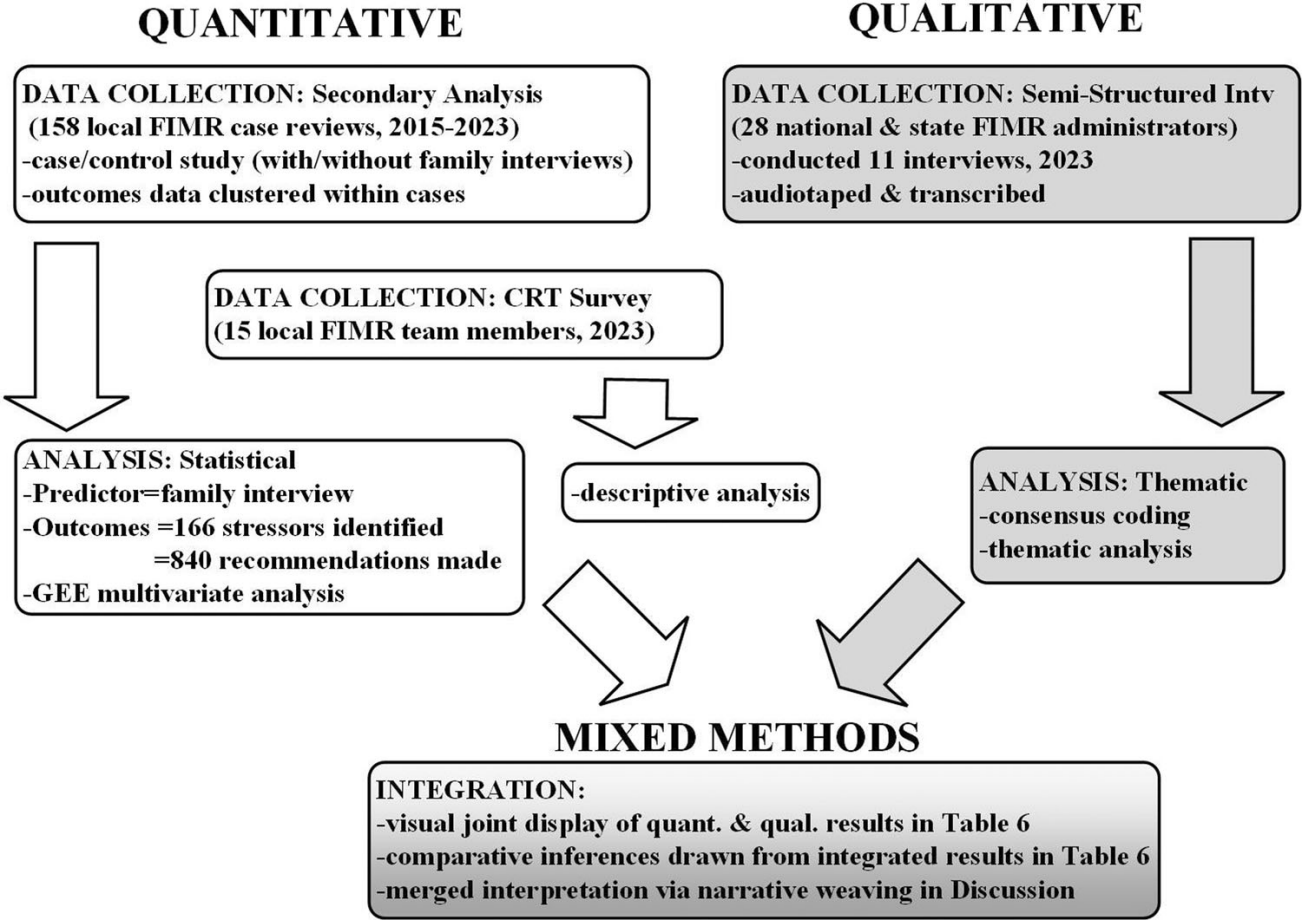
Study parallel convergent mixed‐methods design.

The type of mixed‐methods design used in the present study was parallel convergent design, where quantitative and qualitative data are collected and analysed concurrently to inform a central question [13]. As depicted in Figure 1, quantitative and qualitative data were collected and analysed separately and then integrated using mixed‐methods best practices: visual joint display of results, comparative inferences and narrative weaving within the discussion [13, 14, 15]. This study included quantitative data from FIMR case review administrative records and the local annual FIMR evaluation survey, and qualitative data from semi‐structured interviews with national and state FIMR administrators.

The quantitative study setting was Kalamazoo County, Michigan, characterized by infant mortality rates comparable to the nation (*z* = 0.816, *p* = 0.41), as shown in Table 1. Kalamazoo County has higher Black–White disparities than the nation and the state of Michigan; due to a combination of lower White rates (national comparison: *z* = 5.830, *p* < 0.001; state comparison: *z* = 5.026, *p* < 0.001) and higher Black rates (national comparison: *z* = 5.206, *p* < 0.001) (https://www.mdch.state.mi.us/osr/chi/InDx/frame.html, accessed 1/2/2024).

**Table 1:** National, state, and county infant mortality rates (IMR) by race.

	2021 IMR				Three‐year moving average IMR, 2019–2021^a^	
	US IMR	[Deaths]/[births]	Michigan IMR	[Deaths]/[births]	Kalamazoo county IMR	Total 3‐year [deaths]/[births]
Total	5.4	[19,928]/[3,664,292]	6.2 ± 0.5	[656]/[105,022]	5.2 ± 1.5	[44]/[8522]
Race						
Non‐Hispanic White	4.4	[19,928]/[1,887,656]	4.2 ± 0.5	[317]/[76,190]	2.9 ± 1.3	[19]/[6368]^b^
Non‐Hispanic Black	10.6	[5463]/[517,889]	15.5 ± 1.8	[285]/[18,384]	14.6 ± 5.7	[23]/[1612]^b^
Non‐Hispanic Native Hawaii/Pacific Island	7.8	[74]/[9531]	6.5 ± 2.6	[24]/[3702]	c	c
Non‐Hispanic Asian	3.7	[788]/[213,813]				
Non‐Hispanic American Indian/Alaska Native	7.5	[195]/[26,124]	11.1 ± 7.2	[9]/[808]	c	c
Hispanic ethnicity	4.8	[4246]/[885,916]	7.9 ± 2.1	[56]/[7056]	c	c

*Note:* Infant mortality rate (IMR): Computed by dividing the number of infant deaths in a calendar year by the number of live births registered for the same period. IMR is the most widely used index for measuring the risk of dying during the first year of life. Adding and subtracting the number shown after the ± symbol from the rate creates a confidence interval indicating that the true rate lies between the lower and upper bounds of this interval with 95% statistical confidence.

a https://www.mdch.state.mi.us/osr/chi/InDx/frame.html (accessed January 2, 2024).

b Calculated by clk, using vital records infant death coort files and birth files.

^c^ Data suppressed if cell count < 6 to preserve confidentiality.

Funded through multiple public and private grants, family interviews have been a central feature of Kalamazoo FIMR since its inception in the mid‐1990s. Initially led by obstetric and midwifery providers, the study settings' FIMR programme played a historically formative role in infant mortality prevention efforts at the local and state level, providing FIMR programme templates and conducting trainings across the state [16].

**FIMR family interview.** The study's current FIMR programme and family interview protocol follow national guidelines (https://ncfrp.org/wp-content/uploads/FIMR_Parental_Interview_Guidance.pdf). Specifically:

1. Family contact information is obtained via public records and from abstracted records.
2. Introductory letter is mailed to family, expressing condolence for their loss and explaining the FIMR programme.
3. Phone contact is then made to answer questions about FIMR and the interview, ending with an invitation participate. It is emphasized that this is completely voluntary and that their confidentiality (their identities) will be protected.
4. If agreed, then a face to face meeting is scheduled, at a location and time of the family's choosing. If not interested, then a bereavement resource packet is mailed to them.
5. Interviewers prepare by assembling the following materials in a lockbox:
  o Names of infant and parents, address of parents.
  o Interviewer's phone (on silent), a digital audio recorder, the interview consent and script, bereavement support resource list and the interviewer's business card
6. Upon arrival at the interview location, the interviewer reviews the purpose of FIMR, how the interview will be conducted and the consent form. Then, the parents are invited to sign a consent form and indicate if they give permission for the interview to be audio taped.
  o It is up to the parents whether they would like individual interviews in private or would like their family or friend present.
  o Interviewers also explain that they are mandated reporters. If the interviewer suspects abuse of the deceased infant or observes abuse or neglect of surviving children in the home, it must be reported to child welfare.
7. After the interview, the family is offered the bereavement resource packet and asked for permission for the interviewer to check back in with them in a few weeks. They also receive a $30 gift card to reimburse them for any expenses they incurred as a result of participating in the interview.
8. The interviewer goes directly to the FIMR office to secure the signed consent and any documents with identifiers in the locked FIMR cabinet and to upload the completed interview audiotape onto a secure sharedrive.
9. Within the following 2 weeks, the interviewer will transcribe the interview. Relevant quotes will be abstracted, deidentified and integrated into the narrative case summary.

The family interviewers are professionals with public health, social work and/or counselling expertise. They receive additional training in the cycles of grief, conducting interviews and providing resource referrals for families. As part of their training, they roleplay the interview with a mentor, a current family interviewer on the team, then they shadow their mentor for one or two interviews before conducting their own independently. Once on their own, they check in with their mentors, as questions arise.

### 2.2 Quantitative Methodology

The quantitative component utilized a case‐control design comparing the outcomes of case reviews with a family interview and those without. The study sample was the entire population of 158 stillbirth and infant death reviews completed by Kalamazoo County FIMR during the study period, 2015–2023. Given that multiple findings and recommendations were generated for each case reviewed, statistical analysis was conducted at the case level (*N* = 158), as well as the item level (*N* = 166 findings [contributing stressors] and *N* = 840 recommendations generated). Data collection was secondary analysis of integrated administrative records from National Fatality Review Case Reporting System (NFR‐CRS) case findings, REDCap‐entered case birth and death certificate data, REDCap‐entered family interview tracking records and Kalamazoo‐developed FIMR Action Recommendations. Given the higher infant mortality experienced by Black people across the nation and within the study site, maternal race was included as an important potential confounder of family interview completion and FIMR outcomes. Race, self‐reported on birth certificates, was operationalized to include multi‐raciality, as prior study site research identified that multi‐raciality among Black, Latino, Asian, Middle Eastern and Native American peoples was a significant predictor of birth outcomes and infant mortality [17].

Case findings were measured using the NFR‐CRS Life Stressors items (https://ncfrp.org/wp-content/uploads/DataDictionary_CRS_v6-0.pdf). The Life Stressors items, recently added to NFR‐CRS, captured the contextualized experiences of the family in socioeconomic, medical service and family relationship categories. Case recommendations were measured using the Kalamazoo FIMR Action Recommendations data set, which was developed as part of an effort to translate case review findings into practice and policy. Initiated in 2019 by Kalamazoo FIMR, this 5‐year multiphase process of quantifying FIMR recommendations led to 99 codes within eight categories (see Appendix 1 for process timeline and Table 2 for coding details), which are as follows:

- Improve Systems of Care
- Strengthen Medical Provider Communication
- Family and Community Education
- Address Social Risk
- Care Coordination
- Person‐Centred Decision‐Making
- Bereavement
- Integrate FIMR into Policy/Process Improvement

**Table 2:** Kalamazoo FIMR Action Recommendation categories and codes (*N* = 840 recommendations).

**Improve Systems of Care (health promotion across and within systems) 20.7% (174)**
Create and improve interconceptional care—prepping for next pregnancy, contraception, spacing (including adolescents)
Implement practice‐ and evidence‐based guidelines in medical and social care
Maximize insurance coverage/use of benefits
Develop grassroots and multi‐system response to Safe Sleep issues
Provide parents with paid maternity/paternity or bereavement leave (bereavement or advocacy)
Improve access, availability and affordability of quality child care (as a system of care)
Develop holistic response for failure‐to‐thrive infants (nutrition and weight gain education, resource, monitoring)
Address real‐life barriers/beliefs that prevent families from implementing recommended practices
Organize care for the convenience of families
Continuity of care (same provider/set of providers)
Automate linkage from early pregnancy testing sites to prenatal care
Automate linkage of emergency departments to primary care practices (including obstetric)/case manager/home visitation for pregnant women
Streamline service delivery—medical, social (make care utilization as easy as possible for patient)
Supersize version of system wraparound for highest risk families (e.g., Targeted Universalism)
Cultivate a culture/environment/care plans that support equity, respect for persons, empathy, trauma‐informed care
Maximize insurance coverage/Use of benefits
**Strengthen Medical Provider Communication (skill development) 18.6% (156)**
Improve receptiveness of providers (assist patients, educate providers, implement processes—listening to patients, respect)
Develop provider skills for real conversations, shared decision‐making, relationship building with patients
Ensure consistency—and sharing—of information (across shifts, across providers, inter‐professional, multidisciplinary)
Improve emergency medical response/administrative records documentation, ease of usage, interoperability (including important info on page 1)
Provider—include both parents/identified support partner(s) in the discussion of options and delivery of education
Implement policies that promote debriefing and care for compassion burnout, tools for self‐care for staff
Use multidisciplinary team approach (inside and outside org) especially when multiple systems involved and families are stretched
Education/teams/workshops regarding cultural competency, anti racism, trauma informed care and person‐first language
Direct access to provider or team member (including use of telehealth)
**Family and Community Education/communication (content, process) 17.4% (146)**
Provide appropriate and accessible educational materials (reading level, language, content, appropriate pace and timing)
Services provide adequate resources for translation/addressing language/cultural barriers in all aspects of care
Include both parents/identified support partner(s) in the discussion of options and delivery of education
Develop processes for checking patient understanding of issues, outcomes and their options (especially when bad news)
Improve education of non‐provider healthcare staff and other responding systems (social service, public health, emergency medical system, etc)
Offer second opinions/consults
Institute repetitive messaging for key/emotional medical issues
Preventive Education regarding: happy baby, trauma, development, safety (for parents and support people)
Parenting education regarding: what is acceptable care
Ensure consistent messaging, social marketing for emerging/intractable trends
Provide content training, harm reduction approach on emerging risks and available resources (to providers, community, patient)
**Address Social Risk (system‐level strategies to address individual‐level risk) 15.1% (127)**
Increase capacity to serve more individuals, for longer
Automate screening of material, social and psychosocial risk (at multiple times)
Automate education/linkage to resources
Use evidence based approaches to identify, address social risk (ex neonatal abstinence syndrome protocol, domestic violence, etc)
Improve ease of identification of ALL available/eligible services (Resource first, direct link to services)
Implement risk‐stratified response
Refer all at‐risk individuals/families/populations to community health work/home visitation services (including universal consent, warm handoff)
Implement intentional recruitment practices (hiring of people of colour, people from community [pipeline])
Institute a Housing safety net
Facilitate access to foster care/respite services for individuals/families
Meet people where they are (location, schedule, priorities, literacy, socially, etc)
Support counselling options
Provide transportation resources (including child car seat) and education
**Care Coordination—programme level (between and within medical and community systems) 11.5% (97)**
Implement and/or develop case management and navigation services
Close loop on referrals
Increase street outreach
Maximize utilization of existing resources and services (e.g., perinatal mental health)
Improve ease of pharmacology consult (regarding medication interactions, dosage, etc)—for providers, patients and family
Map existing processes/access population databases/patient input to identify gaps, resources
Coordinate with criminal justice and social system (criminal justice, child protection services, medical examiner)
Establish step‐down hand‐offs between child protection services and maternal‐infant programmes
Invest in child protection worker training, support, supervision
Co‐location of services
Systematic follow‐up for missed appts
Ensure coordination/appropriate handoff among medical providers
**Person‐Centred Decision‐making (strategies) 6.7% (56)**
Integrate doulas into care continuum (advocacy, information, empowerment, loss)
Create menu of care options (centring, bundled mother/baby visits)
Empower patients to look at accurate, high‐quality websites/other resources
Explicitly integrate patient religious/cultural preferences into decision‐making
Engage fathers/partners/caregivers in decision‐making
Empower patient questions/requests/decision‐making
Explain all treatment options, including risk/benefits
**Bereavement (resources, services) 5.2% (44)**
Develop a coordinated bereavement network for linking families to resources + follow‐up (including Fetal Infant Mortality Review role)
Maintain menu of resource options (counselling, group, family, support, etc)
Maintain updated list of bereavement resources
Publicize list with medical, funeral homes, etc
Include help with funeral arrangements/costs with follow‐up
Include all types of perinatal loss
Provide emotional support to families during crisis (allowing time, skin contact, etc)/forensic investigation
Engage partners/caregivers/siblings throughout the bereavement process
Primary care practices create coordinated response to loss, questions, reassurance
**Integrate Fetal Infant Mortality Review into Policy/Process Improvement Efforts 4.8% (40)**
Establish pipeline for Fetal Infant Mortality Review recommendations to reach medical and public systems
Create avenue for patients/families to communicate with medical administrators
Establish process for stakeholder prioritization of FIMR identified issues/recommendations
Bring in specialists to educate Fetal Infant Mortality Review and to learn from Fetal Infant Mortality Review (breast feeding, child care, pharmacists, police, etc)
Include father of baby in interview/records review/narrative
Document key health education in records
Improve Fetal Infant Mortality Review data access/collection (interview, administrative records)
Explicitly identify what DID work (strengths based)
Recommend/use epidemiology study of issue
Recommendations for population health

*Note:* Categories are rank‐ordered from highest to lowest counts, whereas codes are not rank‐ordered.

As noted previously, the multiple responses for case findings and case recommendation were analysed at both the case level and the findings and recommendations item level. At the case level, measures were aggregated, by category, into multinomial and count variables. The recent addition of the Life Stressors section to NFR‐CRS meant that only 66 of the 158 cases had data entered; the number of stressors identified per case ranged from 0 to 12, with 166 stressors identified in total. The Life Stressor categorical variable sorted data into four categories: no stressor identified, socioeconomic stressor, medical service stressor and family relationship stressor. Recommendations data were available for all 158 cases, with 1–18 recommendations made per case, and a total of 840 recommendations. Each recommendation was categorized into one of eight category foci: systems improvement, provider communication, family/community education, addressing social risk, care coordination, person‐centred decision‐making, bereavement, and integrating FIMR process/recommendations.

To address concerns related to the smaller, subset sample with Life Stressor data (*n* = 66), two analyses were conducted: (1) bivariate comparison of infant death and maternal demographic characteristics between the 66 cases with stressor data and the 92 cases that were entered in earlier NFR‐CRS database versions without a stressor data section (Appendix 2); and (2) bivariate comparison of NFR‐CRS Issue responses from the family interview group (*n* = 53) within the full sample and those from the family interview group (*n* = 15) within the Life Stressor subset (Appendix 3). For this latter analysis, the NFR‐CRS Issues outcomes were selected as they contain comparable content categories (maternal medical, substance use, social support, etc, https://ncfrp.org/wp-content/uploads/DataDictionary_CRS_v6-0.pdf) that were entered for all cases. Neither analysis found statistical differences (*p* values were all above 0.05, indicating that the comparison groups were statistically similar). Furthermore, the absolute differences between full sample and subset family interview groups across the 17 NCFRS Issue categories averaged modest seven percentage points, indicating that, for all practical purposes, the two groups appear similar.

Pearson's chi‐squared, Fisher's exact (for expected cell counts < 5) and ANOVA statistics were used for bivariate comparisons of cases with and without family interviews. A range of medical, healthcare and demographic characteristics were considered as potential confounders (reported in Table 3) including whether infant or stillbirth death, age at death (for infants), cause and manner of death (for infants), birthweight and gestation (for infants), whether single‐ or multi‐gestation, maternal age, race and ethnicity, maternal education, insurance status at the time of birth, perinatal health care and service referrals. Generalized Estimating Equation (GEE) SPSSv.29 was selected for the multivariate analysis to account for the non‐independent nature of the data, as multiple findings and recommendations were clustered within each case. The independent variable was presence or absence of family interview, a dichotomous variable, where the absence of a family interview served as the reference. Four outcomes were modelled: (1) item‐level category of Life Stressor content and (2) of Recommendations content, (3) case‐level counts for number of Life Stressors identified (total as well as by category) and (4) number of Recommendations generated (total as well as by category). Authors selected the multinomial regression model for item‐level analysis of the two categorical dependent variables (Life Stressor and Recommendations content categories). Reference categories for each of the dependent variables were selected based upon their conceptual distinctiveness [18], ‘no stressors’ was the reference category for Life Stressors and ‘integrating FIMR’, a FIMR‐administrative category, was the reference category for Recommendations. GEE Poisson loglinear modelling was selected for both count outcomes. Multivariate regressions adjusted for covariates ‘death type’ and ‘death manner’. An independent correlation structure was used, which assumed that, while observations within clusters were correlated, the clusters themselves were independent [19]. Sensitivity analyses were conducted with autoregressive and exchangeable correlation structures, which did not substantively change parameter estimates [19], confirming the independence assumption and indicating robustness of model structures. Threshold for statistical significance was two‐sided and set at *α* 0.05.

**Table 3:** Bivariate comparison of cases with and without family interviews (row percentage).

	Family interview (*n* = 53) % (count)	No nterview (*n* = 105) % (count)	*p*
**Death category**			< 0.001
Infant death (< 1 year)	27% (35)	73% (97)	
Stillbirth death^a^	69% (18)	31% (8)	
**Infant age at death (infants only)**	(*n* = 35 infants)	(*n* = 97 infants)	
Age in weeks (mean)	7.1 weeks	7.2 weeks	0.95
**Infant death cause category(infants only)**	(*n* = 35 infants)	(*n* = 97 infants)	0.07
Prematurity	23% (11)	77% (36)	
SUID/sleep‐related	12% (4)	88% (29)	
Congenital anomalies	33% (9)	67% (18)	
Infection/disease	55% (6)	45% (5)	
Complications (pregnancy/delivery)	30% (3)	70% (7)	
Homicide	b	b	
Accident (non‐sleep‐related)	(0)	b	
**Infant death manner (infants only)**	(*n* = 35 infants)	(*n* = 97 infants)	0.03
Natural	31% (29)	70% (66)	
Accident	b	88% (30)	
Homicide	b	b	
**Infant characteristics (infants only)**			
Birthweight, grams (mean)	1526 g	1596 g	0.75
Gestation, weeks (mean) [missing]	29.3 weeks	30.9 weeks [3]	0.36
**Pregnancy characteristics(infants only)**			0.72
Single gestation	34% (47)	66% (91)	
Multiple gestation	30% (6)	70% (14)	
**Perinatal care [missing]**	34% (44) **[8]**	**66% (84) [13]**	**0.27**
Preconceptional care [missing]	39% (32) [13]	61% (50) [27]	0.08
Postpartum visit [missing]	37% (39) [9]	63% (66) [19]	0.10
Pregnancy planning [missing]	36% (36) [12]	64% (65) [29]	0.73
Bereavement referral [missing]	35% (39) [12]	65% (74) [17]	1.00
**Maternal demographics (infants only)**			
Maternal age (mean)	28.5 years	28.1 years	0.64
Maternal race category			0.62
White only	38% (29)	62% (48)	
Black (only, multi‐race)	28% (19)	72% (49)	
Latina (only, multi‐race)	b	b	
Middle East. (only, multi‐race)	b	b	
Native Am. (only, multi‐race)	b	b	
Asian (only, multi‐race)	0	b	
Maternal insurance status			0.13
Medicaid	32% (28)	68% (59)	
Private insurance	40% (25)	60% (38)	
Self‐pay/other [missing]	0	100% (6) [2]	
Maternal Education			0.05
< High School	29% (8)	71% (20)	
High School degree	35% (18)	65% (34)	
Some college	27% (9)	73% (24)	
Associate degree	0	100% (7)	
Bachelor degree	61% (14)	39% (16)	
Masters degree	b	67% (6)	
Doctorate/Prof degree [missing]	0 [1]	b	
**Service referrals [missing]**	[21]	[35]	
Any referrals (valid %)	31% (25)	69% (55)	0.96
Average number of referrals (mean)	2.6 referrals	2.9 referrals	0.45

a 22+ weeks gestation and > 450 g at birth, no sign of life at birth [MI Stillbirth Registry].

b Data suppressed if cell count < 6 to preserve confidentiality.

Since 2015, an annual evaluation of the Kalamazoo County FIMR case review team (CRT) process has been conducted. All individuals who have participated in at least one case review meeting in the prior year are invited to participate. Questions typically include demographics, profession, and the ways that participating in FIMR has impacted their work practices. The evaluation is conducted anonymously using RedCap survey QR code link. In 2023, additional questions were added to elicit CRT members' experiences with family interviews. Two quantitative questions (5‐point Likert scale) asked CRT members about the impact of family interviews ‘… upon your ability to effectively identify gaps within systems of care?’ and ‘… upon your ability to effectively produce recommendations’. An additional open‐ended question asked them to ‘… explain your responses regarding the impact of family interviews’. All 22 individuals who had attended a FIMR meeting the prior year were invited to complete the 2023 evaluation survey, and 15 completed them (68% response rate). Given the small sample size, only descriptive analyses (response counts and percentages, with explanatory comments) were conducted.

### 2.3 Qualitative Methodology: Semi‐Structured Interviews

With the goal of triangulating and extending quantitative data, semi‐structured individual and group interviews were conducted with FIMR administrators across the United States regarding their experiences with family interviews. The qualitative study sampling method was purposive, including 28 current and former FIMR administrators. Data collection consisted of 11 semi‐structured interviews: individual interviews with 15 national FIMR administrators, and a group interview with 13 Michigan FIMR Network members. Most interviews were completed virtually using MSTeams. Two participants were interviewed face to face, and two participants submitted written responses to interview questions. Interviews were audio‐taped and transcribed. Independent coders (F.O. and N.E.) separately analysed transcripts, using MaxQDA, to (1) identify initial themes [20] and (2) further analyse and code quotes. Coders used consensus to finalize and refine themes and sub‐themes.

## 3 Results

### 3.1 Quantitative Results

#### 3.1.1 Family Interviews

A third (34%, *n* = 53) of the 158 cases reviewed included a family interview. Of the remaining 105 cases without interviews, the majority (66%, *n* = 69) were lost to contact, whereas 34% (*n* = 36) were declined by the families. Cases obtaining family interviews were not statistically different from those without interviews regarding multiple perinatal characteristics, including infant characteristics (age at death, birthweight and gestational age), pregnancy characteristics (multi‐gestation or not), perinatal healthcare and social service referrals and maternal demographics (age, race and ethnicity, insurance status, education) (Table 3). Likewise, among the non‐interview cases, cases where families were reached but declined were comparable to those where they were never reached (Appendix 4). Notable differences were that higher interview rates were associated with stillbirth deaths (69% vs. 27% infant death, *p* < 0.001), and with homicide (67%) and natural (31%) deaths over accidental deaths (12%, *p* = 0.03). The higher stillbirth interview rate likely stemmed from a protocol change made in 2019 where the only stillbirth cases that made it into the case review process were those that had completed interviews; unlike infant death cases where all were reviewed regardless of completed family interview. To account for potential confounding by pre‐existing case differences, subsequent multivariate modelling controlled for them by including death type and manner variables in the model. As the goal was to isolate the family interview effect rather than that of pre‐existing differences, the GEE multivariate regression results in Tables 4 and 5 reported post‐intervention (family interview) estimates only, mentioning in a footnote that the model ‘adjusted for death type and manner’.

#### 3.1.2 Review Findings

The case review findings' measure (NCF‐CRS Life Stressors), controlling for death type and manner, produced a statistically significant association between family interviews and identification of life stressors (Table 4). Family interviews were significantly associated with the number of stressors identified as well as the types of stressors documented during case reviews. When family interviews were present, the average number of stressors recorded more than doubled, from a mean of 1.6 stressors to a mean of 4.1 stressors (Table 4, case‐level descriptive statistics). GEE Poisson loglinear regression results confirmed this relationship, indicating a 2.6 increase in the number of stressors identified when a family interview was present (aOR = 2.6, 95% CI: 1.5–4.7, *p* < 0.001, a Goodness of fit QICC measure of 166.3 in Table 4, case‐level multivariate regression results). Additionally, the presence of a family interview increased the odds that medical service‐related stressors, such as inaccessible services and cultural barriers to care, were identified in the case review process, as detailed across all medical service‐stressor measures reported in Table 4. Item‐level results show that 47.6% of family interview case reviews identified medical service stressors, compared to 27.7% of case reviews without an interview, increasing the odds by 17.9 that, compared to zero stressors identified, a medical service stressor would be found (aOR = 17.9, 95% CI: 3.3–96.6, *p* = 0.003, Goodness of fit QICC = 210.0). Likewise, case‐level results show that the presence of a family interview quadrupled the average number of medical service stressors identified, from a mean of 0.5 to a mean of 2.0 (aOR = 3.9, 95% CI: 2.1–7.4, *p* < 0.001, Goodness of fit QICC = 86.4). Finally, although the overall prevalence of socioeconomic stressors identified didn't vary based upon family interview, the average number did. Among case reviews identifying any socioeconomic stressor, such as poverty and discrimination, the presence of a family interview was associated with twice as many being identified compared to cases without an interview, from a mean of 0.9 socioeconomic stressors to a mean of 1.9, confirmed by the GEE results of a 2.3 increase (aOR = 2.3, 95% CI: 1.1–4.8, *p* = 0.03, Goodness of fit QICC = 132.2).

**Table 4:** Impact of family interview upon the CRT Life Stressors identified.

	Descriptive statistics^a^				GEE multivariate regression, with interview (vs. no interview)							
	Item level (144 stressor items endorsed)		Case level (*N* = 66) Mean number		Item level (144 stressor items endorsed) Multinomial models (categorical data)				Case level^b^ (*N* = 66) Poisson models (count data)			
	Interview (61 stressors) % ‘yes’	No interview (83 stressors) % ‘yes’	Interview (15 cases) (95% CI)	No interview (51 cases) (95% CI)	aOR	(95% CI)	*p* a	Goodnes of fit QICC	aOR	(95% CI)	*p*	Goodness of fit QICC
**Total**	**100%**	**100%**	**4.1 (2.7, 5.5)**	**1.6 (1.1, 2.2)**					**2.6**	**(1.5, 4.7)**	< **0.001**	**166.3**
Socioeconomic‐related (75 items endorsed)	44.4% (28)	56.6% (47)	1.9 (0.9, 2.8)	0.9 (0.2, 0.6)	2.2	(0.6, 8.8)	0.50	210.0	2.3	(1.1, 4.8)	0.03	132.2
Medical service‐related (53 items endorsed)	47.6% (30)	27.7% (23)	2.0 (1.3, 2.7)	0.5 (0.3, 0.7)	17.9	(3.3, 96.6)	0.003		3.9	(2.1, 7.4)	< 0.001	86.4
Relationship‐related (16 items endorsed)	4.8% (3)	15.7% (13)	0.2 (0.0, 0.4)	0.2 (0.1, 0.4)	0.6	(0.1, 3.2)	0.55		1.0	(0.3, 3.4)	0.99	59.4
No stressors (22 cases with zeros for all items)	2 cases	20 cases	not applicable		[reference]				not applicable			

a Bootstrapping results based upon 1000 bootstrap samples.

^b^ Adjusted for death type and manner.

#### 3.1.3 Recommendations

Most case reviews generated multiple recommendations, up to 18 for a single case. Applying the Kalamazoo FIMR Action Recommendations coding scheme to the 158 study case reviews generated 840 primary recommendation codes (when a recommendation met criteria for multiple codes, a primary code was identified). Just as with the stressor findings above, family interviews were significantly associated with both the number and the types of recommendations generated during case reviews. When family interviews were present, the average number of total recommendations rose from a mean of 4.9 recommendations to a mean of 6.1 (Table 5, case‐level descriptive statistics). GEE Poisson loglinear regression results confirmed this relationship, indicating a 40% increase in the number of recommendations produced when a family interview was present (aOR = 1.4, 95% CI: 1.0–2.0, *p* = 0.05, a Goodness of fit QICC measure of 102.7 in Table 5, case‐level multivariate regression results). Additionally, the presence of a family interview increased the odds that provider communication recommendations, such as improving provider shared decision‐making skills and provider cultural‐competency (see Table 2 for itemized list), were identified in the case review process. Item‐level results show that 23.0% of family interview case reviews generated provider‐communication recommendations, compared to 15.8% of case reviews without an interview. Likewise, case‐level results show that the presence of a family interview doubled the average number of provider communication recommendations produced, from a mean of 0.8 to a mean of 1.4 (aOR = 2.2, 95% CI: 1.1–4.4, *p* = 0.02, Goodness of fit QICC = 121.7). Additionally, making bereavement recommendations was significantly more likely to occur if a family interview was present than if it was not, increasing in likelihood by a factor of 5, from 2.9% to 9.0% (aOR = 5.4, 95% CI: 1.6–18.4, *p* = 0.05, Goodness of fit QICC = 573.7).

**Table 5:** Impact of family interview upon the CRT recommendations generated.

	Descriptive statistics^a^				GEE multivariate regression, with interview (vs. no interview)							
	Item level (840 recommendations)		Case level (*N* = 66) Mean number		Item level (840 recommendations) Multinomial model (categorical data)				Case level^b^ (*N* = 158) Poisson models (count data)			
	Interview (322 rec'd) % ‘yes’	No interview (518 rec'd) % ‘yes’	Interview (53 cases) (95% CI)	No interview (105 cases) (95% CI)	aOR	(95% CI)	*p* a	Goodnes of fit QICC	aOR	(95% CI)	*p*	Goodness of fit QICC
**Total**	**100%**	**100%**	**6.1 (5.0, 7.2)**	**4.9 (3.4, 5.5)**					**1.4**	**(1.0, 2.0)**	**0.05**	**102.7**
Improve Systems (174 recommendations)	20.5% (66)	20.8% (108)	1.3 (0.8, 1.7)	1.0 (0.8, 1.3)	3.3	(0.9, 12.7)	0.40	573.7	1.1	(0.6, 2.1)	0.75	85.2
Provider Communication (156 recommenddations)	23.0% (74)	15.8% (92)	1.4 (0.9, 1.9)	0.8 (0.6, 1.0)	1.6	(0.4, 6.8)	1.00		2.2	(1.1, 4.4)	0.02	121.7
Family/Community Education (136 recommendations)	16.8% (54)	17.8% (82)	1.0 (0.8, 1.3)	0.9 (0.7, 1.1)	3.9	(1.0, 14.8)	0.30		1.7	(0.9, 3.3)	0.09	111.9
Address Social Risk (127 recommenddations)	11.5% (37)	17.4% (90)	0.7 (0.4, 1.0)	0.9, 0.7, 1.1)	0.7	(0.2, 2.6)	1.00		1.0	(0.4, 2.3)	0.98	107.1
Care Coordination (97 recommenddations)	7.1% (23)	14.3% (74)	0.4 (0.3, 0.6)	0.7 (0.5, 0.9)	0.8	(0.2, 2.8)	0.70		0.8	(0.4, 1.7)	0.55	91.1
Person‐Centred Decision‐Making (56 recommendations)	8.7% (28)	5.4% (28)	0.5 (0.4, 0.7)	0.3 (0.2, 0.4)	2.2	0.7, 7.1)	0.40		1.2	(0.5, 2.8)	0.64	63.6
Bereavement (44 recommenddations)	9.0% (29)	2.9% (15)	0.6 (0.3, 0.9)	0.1 (‐.1, 0.2)	5.4	(1.6, 18.4)	.05		2.2	(0.6, 7.7)	0.23	71.5
Integrate FIMR (40 recommendations)	3.4% (11)	5.6% (29)	0.2 (0.1, 0.3)	0.3 (0.2, 0.4)	—				0.9	(0.2, 4.2)	0.89	40.2

a Bootstrapping results based upon 1000 bootstrap samples.

^b^ Adjusted for death type and manner.

#### 3.1.4 Evaluation Survey Results

The 15 Kalamazoo County CRT members completing annual evaluations attended an average of six (of 11) 2023 FIMR meetings. Over half (8 of 15, 53%) indicated that they would initiate changes as a result of FIMR (institutional policy and their own practice). The majority (10 of 15, 67%) reported that family interviews were Very Impactful on their ability to ‘effectively identify gaps within systems of care’, as illustrated in an evaluation respondent's explanatory comment below:

Without their perspective, we don't have the full story of what happened and why gaps may have occurred.

(Respondent #11)

The same number, 67%, rated family interviews Very Impactful on team members' ability to ‘effectively produce recommendations’ and make meaningful systems' change:

They [family interviews] provide a new perspective on the healthcare system and what needs to be adjusted …

(Respondent #2)

The workplan my organization is based off of stems from the FIMR recommendations, meaning we take personal experiences into consideration when deciding what issues need to be addressed in our community

(Respondent #14)

### 3.2 Qualitative Results

#### 3.2.1 Conducting Family Interviews

The central theme emerging from qualitative study participants' discussions regarding obtaining family interviews was *Hurdles to Getting Interviews*, as illustrated in the following quote:

We just, we struggle mightily to get interviews with our families, and we do, we make multiple connections, handwritten cards, calls ….

(Interview #10)

Qualitative study participants offered ideas for overcoming obstacles, including reducing the time between death and contact, utilizing social network connections, creating safe spaces to share stories and using a more relational approach.

Sometimes it's the way we say things to those families to make those impressions. So, being very approachable, making it feel like it's a conversation, like you care …

(Interview #10)

#### 3.2.2 Findings Identifying Gaps

The predominant qualitative theme related to the impact of family interviews upon the FIMR process was *Completing the Picture*, where family interviews contextualize the events and circumstances surrounding their care and the death itself. As illustrated in Table 6, qualitative study participants found that the presence of interviews allowed a more robust, comprehensive review, effectively reducing bias and challenging the presumed objectivity of records' documentation.

**Table 6:** Qualitative themes, sub‐themes, illustrative quotations and sources.

Sub‐Themes	Exemplar quotes	Data source
**Theme: ‘Family‐Interviews Complete the Picture’**		
Providing more complete case information	‘Oh, I think without a maternal interview, in my opinion, there is no way to have a complete picture of the case’.	Qualitative study participant #5
	‘… the medical records are kind of black and white and the interview adds color to the picture’.	Qualitative study participant #1
	‘It just represents to people a deeper understanding of what the story is behind the numbers as well as the connection or the lack of a connection made with certain providers and potential gaps in care…’	Qualitative study participant #13
	‘And then when you ask the family, “So what did they say was the cause of your stillbirth, or the reason your baby passed away, or what was going on during your pregnancy?” And their answers are often not exactly the same. Or you can tell that they didn't fully understand the information that they were given’.	Qualitative study participant #11
Elucidating social determinants of health	‘It just enriches the entire case to have that extra data. Especially if there's anything related to social determinants of health, because that's really not reflected in the medical records all the time’.	Qualitative study participant #13
	‘It's very different from reading someone's like medical records that are in the perspective of their doctors and their medical care team versus marrying the more emotional aspect, more of the social determinants that they're living with every day’.	Qualitative study participant #7
Facilitating a more focused case review	‘… direct quotes are helpful for [case review team members] to like, gain a better understanding of. what was going on like in that moment for, for the family. So, I think that that can be impactful with like recommendations that that we're making especially with like social determinants of health, not maybe in as much with like the medical side of things, but maybe what other resources or like uh, mental health care could have been provided for that mom or their family in the moment’.	Qualitative study participant #2
	‘… it keeps it from going off the rails. I think a little bit and that makes sense because there is more concrete information that we have from the family instead of the “what ifs”’.	Qualitative study participant #6
Undercutting bias and blame	‘To that point, I think it gives people perspective like oftentimes we see in a medical record that a patient is “noncompliant” with their diabetes or their high blood pressure or anything like that. Whereas if we talked to someone and you know they say “Oh, I have trouble managing my blood sugar because I work three jobs and it's hard to provide myself with nutritious food and check my blood sugar in between my shifts …. So I think it gives us perspective as well because you know if you see a patient's not compliant you probably you know even if you try to remain unbiased you know those words themselves have carry a stigma with them’.	Qualitative study participant #1
	‘I think that these interviews sometimes can help you see all the things that people are up against to maybe be a little bit more empathetic or to not look so much at the individual, but to step back and see the things that may be could help instead of just personal decisions and to kind of think more systemically and not be as critical, I think sometimes’.	Qualitative study participant #6
	‘… I feel like I've been in those spaces in the CRT where clinical folks might comment. And there's some sort of like, unfortunately, like judgment lay down because there is no information from the family there. There's no family voice, so I feel like it's a different it's an entirely different conversation when we have that family voice’.	Qualitative study participant MI #18
Increasing the scope of recommendations	‘And the efficacy of the care provider themselves on you know educating which is part of caring for your patient a lot of times either Mom says, you know, we don't know what happened when it's very clear cut in the in a medical record or they'll explain something very different from what we're reading … UM, and you know, I think if we were able to maybe employ like a teach back method or something like that, maybe the thing, you know, Mom, what Mom tells us would be more in line with what the provider documents in the record’.	Qualitative study participant #1
	‘… some recommendations on a policy level are dependent on hearing the voices and perspectives of these families’.	Qualitative study participant #15
Practical recommendations focused on specific processes for improvement	‘I think that we've had a number of recommendations surrounding grief and like a developing something like a post loss clinic where families can work through the loss itself with the medical team to gain some closure and I don't think without an interview we would ever have sparked that idea because the medical record ends, you know, oftentimes before even a postpartum visit …’	Qualitative study participant #1
	‘And it gives insight into the perspectives of the experience as well, like the way that staff talked to the clients has really had a profound impact on their perceptions. It's not only the experience of the healthcare that's received, but the way they're talked to. Their tone that they use, the words that we choose to use and how they phrase things too. So sometimes some of our recommendations has been to, umm, provide like more sensitivity training to some of the staff at hospitals as well’.	Qualitative study participant #13
**Theme: Family Interviews Bring the Human Connection**		
Establish a personal connection to the case	‘… it humanizes it and allows their perspective to be given …’	Qualitative study participant #6
	‘We get more with when we have an interview and then just like from the emotional standpoint as well,… So just a little bit, I guess just a little bit deeper of a dive into what their like journey was like’.	Qualitative study participant #2
	‘I think it helps just bring like the emotion or like the realness to the cases, because otherwise we would only have that that medical record’.	Qualitative study participant #2
Motivate action and advocacy (for CRT, CAT, professionals, policy makers and public)	‘… you know we might advocate for something because we know it's important. But you really, really tend to advocate for things when it's personal or it feels personal, or you know the person involved. And I think the family‐interviews help lend some of that’.	Qualitative study participant #12
	‘I don't know if that makes any sort of like immediate impact, but the hope of maybe to like just garner some more attention from like someone in the community. Like you might see, see this quote of like how this Mama struggling or how her bereavement has been to like. Just maybe show more interest in in reading more about the report or what kind of recommendations are out there?’	Qualitative study participant #2
	‘I think that has helped push bereavement work when I can say, “Listen, I talked to a mom just last week and she said she came”’.	Qualitative study participant #11
Barriers to action	‘Our biggest barrier is having staff to work on the recommendations… And so it's hard to put things into action when there's so much that needs to be put on the agenda. And then just finding the people who are actually going to take charge of the recommendations and work on them’.	Qualitative study participant MI #19
	‘I think along the same lines, it's some of the things that we would like to have as recommendations are, I don't want to say impossible, but it's just we have so many people that have mental health needs and not nearly enough people in that field especially that are taking Medicaid or, you know, just like things like child care … And these larger issues that we in our CAT team just don't have the ability to solve those problems’.	Qualitative study participant MI #20
	‘… I would say that we don't have the power to change the policies that impact a lot of our recommendations, right? Like when you're talking about childcare, we don't have good resources for it … So we have to figure out that pathway see those that power to impact policy, I think’.	Qualitative study participant MI #18

Abbreviations: CAT, community action team; CRT, case review team.

#### 3.2.3 Recommendations

The theme of ‘Completing the Picture’ extended to the FIMR recommendation stage. Qualitative study participants reflected that family interviews broadened the scope of recommendations, especially regarding patient‐provider interactions, as well as deepening the specificity of recommendations regarding detailed process improvements (Table 6). The third theme emerging from the qualitative study participants was *Bringing the Human Connection* (Table 6). By increasing the emotional impact on reviewers, family interviews shape what reviewers find most important, informing not just recommendations themselves, but their prioritization and the impetus to act on recommendations. Barriers to recommended action, regardless of family interview, were commonly cited and included insufficient resources to fund programming and lack of structure for policymaking.

### 3.3 Mixed‐Methods Integration

At each of the four FIMR process points shown in Table 7, key qualitative and quantitative research study findings corroborate each other, validating and strengthening inferences regarding the value of family interviews in identifying system gaps and producing actionable recommendations. Additionally, for three of the inferences, either the qualitative or the quantitative results extended the scope of study findings, adding information or a perspective that would have been missing if not for the combined methodologies.

**Table 7:** Joint display of mixed‐methods integrated results.

FIMR process	Method		Mixed‐method inference
	Quantitative results	Qualitative results	
Obtaining family intv (FIMR staff)	2/3 cases had no family intv (most lost to contact) (text, Appendix 3)	‘Multiple Hurdles’ (resource and logistical) to securing intvs (text)	Corroborating: Family intvs are difficult to obtain
	Death type and manner, rather than perinatal or demographic differences associated with obtaining family intv (text, Table 3)	Offered ideas for overcoming hurdles (text)	Additional: adapt family intv. strategy to death type and manner
			Additional: strategies to increase intvs
Identifying gaps (case review team)	Family intvs associated with identifying significantly more socioeconomic and medical stressors (text, Table 4)	‘Complete the picture’, identifying what providers/administrators did not know about family social circumstances and care experiences (Table 6)	Corroborating: contextual info from family intv identifies root causes and challenges assumptions
	67% (10 of 15 CRT evaluation respondents) rate family intvs ‘Very Impactful’ in identifying system gaps (text)	‘without [family intv.] we don't have the full story …’ (text)	Corroborating: increasing the value of FIMR for system improvement
Generating recommendations (case review team)	Family intvs associated with generating significantly more recommendations, especially related to provider communication (Table 5, text)	Having the ‘complete picture’ extends the scope of recommendations, with more recommendations that are innovative for systems and pragmatic for families (Table 6) ‘Bringing the human connection’ engages others at the personal level, motivating them to find solutions (Table 6)	Corroborating: family intvs focus problem‐solving and inform action planning
	67% (10 of 15 CRT evaluation respondents) rate family intvs ‘Very Impactful’ in effectively produce recommendations (text)		Corroborating: family intvs build momentum for implementing change
	More recommendations for bereavement network and resources (Table 5, text)	Bereavement is a common gap identified by families (Table 6)	Corroborating: community‐informed bereavement support
Taking action (case review team and community action team)	53% (8 of 15 CRT evaluation respondents) intend to change their own practice or work to change agency policy (text)	‘Bringing the human connection’ motivates action and advocacy (Table 6, text)	Corroborating: family intv activates change at the personal, institutional and policy level
		Lack of resources and decision‐making power pose significant barriers to implementing recommendations (Table 6)	Additional from qual: significant barriers to addressing root causes

Abbreviations: CRT, case review team; intv, interview.

## 4 Discussion

### 4.1 Main Findings

#### 4.1.1 Improving FIMR Findings and Recommendations

This study provides compelling quantitative and qualitative evidence that family interviews serve a central function in identifying system gaps and generating community‐informed, practical solutions. While confirming the front‐end challenges of obtaining the family interviews [12], qualitative study participants emphasize that family interviews are critical for shedding light upon the circumstances behind family behaviours that otherwise may be subject to blame by service providers and review members alike. Quantitative results document the power of family interviews to reveal the underlying degree and nature of stressors faced by families within the medical environment and in their daily lives. Furthermore, study findings show that family interviews are associated with CRTs producing a greater number of innovative recommendations to address these social determinants of health stressors, particularly in the areas of provider communication and bereavement. Reviews that include a family interview transcript produced significantly more recommendations related to medical provider communication (aOR 2.2, 1.4 recommendations with a family interview vs. 0.8 without, Table 5) such as develop provider skills for relationship‐building and shared decision‐making, institute processes that reinforce active listening by providers, and implement multidisciplinary team approach for complex cases (multiple systems involved with family) or when families are particularly stretched (see Table 2 for specific recommendations within this category). Similarly, reviews with a family interview transcript were significantly more likely to generate one or more bereavement‐related recommendations (aOR 5.4, 9.0% with a family interview vs. 2.9% without, Table 5) such as develop a coordinated bereavement network for linking families to resources and for systematic follow‐up, and include all types of perinatal loss within bereavement efforts (see Table 2 for specific recommendations within this category).

A key challenge to overcome is the 15% family interview rates experienced by FIMRs across the United States. Four out of 10 (41%) of US FIMR teams (both those completing family interviews and those unsuccessful in conducting family interviews) cite inaccurate or missing contact information as the largest obstacle they face, followed by lack of funding for this labour‐intensive activity (13% cite), staff discomfort with the interview process (6% cite) and lack of training (5% cite) [12]. Qualitative study results' theme of ‘Hurdles to Getting Interviews’ confirmed the centrality of this problem and endorsed strategies that have proven successful in the United States and elsewhere: immediate initial outreach, extending the abstraction process to encompass updated contact information, partnering with community health workers or home visitors who are skilled and comfortable with sensitive conversations, and nationally available family interview protocols and training [9, 12, 21]. The extramural funding and collaboration with community health workers implemented by the study site FIMR team enabled them to double the national rate (to 34%), reaching traditionally hard‐to‐engage families across the racial and socioeconomic spectrum. The UK's Perinatal Mortality Review Tool has a comprehensive parental engagement process (in‐hospital invitation, key contact assignment, multiple methods for providing input and follow‐up report answering family's concerns) that has led to a 97% parent contact rate and a 55% parent participation rateit [9, 21].

#### 4.1.2 Activating System Improvements

Beyond improving the quality and scope of FIMR outcomes, study findings suggest that family interviews can serve a critical role in activating system improvements. Whether change agents are FIMR reviewers, community action teams, institutional leaders or policymakers, the human connection established through family interviews was cited by qualitative study participants as a key motivating factor. This is further supported by study‐site FIMR evaluations that half of case reviewers plan to instigate changes as a direct result of FIMR, crediting family interviews as having great impact in this process. While such individuals can champion organizational change, without an existing infrastructure ready to translate recommendations into policy, this can be an uphill struggle [22]. Qualitative study participants confirm the substantial challenges FIMR teams face at the back‐end of the process, e.g., implementing the infrastructure and policy recommendations that can stem from family interviews. As one participant noted, ‘we have to figure out that pathway … to impact policy’.

#### 4.1.3 Focus on Healthcare Services

In the meantime, study results highlight that when family voice is included in FIMR, problems and solutions identified often focus upon medical‐service aspects of the healthcare system. Findings and recommendations produced by cases with family interviews are significantly more likely to identify stressors related to medical provider interactions and lead to actionable recommendations related to provider‐communication and decision‐making; issues that are otherwise invisible to providers and institutions themselves. As these are the very areas that policymakers have focused upon to reduce the stark disparities in maternal health, FIMR and family interviews hold promise for contributing actionable solutions [23, 24]. Plentiful evidence exists regarding how health inequities vary across different settings, how unaware individual providers often are and how embedded they are within institutional structures [25, 26]. The community‐based structure of FIMR means that recommended solutions can be multi‐layer, responsive to local context and state and federal circumstances.

### 4.2 Strengths and Limitations

A primary study strength is the mixed‐methods design [14], which allowed us to capture various aspects of the complex process of incorporating family interviews and the ways they add value. Another strength is the alignment of study findings across methods and across study samples; qualitative study participants across the United States agreed that family interviews identify service gaps that are often unseen by providers and healthcare leaders, which parallel quantitative case review results from the study site that more health service gaps and recommendations identified with family interviews compared to without. An additional strength is the demographic similarity of case reviews with a family interview compared to those without, demonstrating their appeal and impact across race, socioeconomic status, perinatal care and birth outcomes.

A study limitation is that quantitative data stemmed from a single site. Although the study community resembled the United States on many demographics, it had higher than normal racial disparities and, unlike many FIMRs, was well‐supported through multiple funding sources. Authors attempted to ameliorate this limitation by eliciting qualitative‐study participant input from FIMR administrators across the nation, who possessed a range of family interview experiences. Another notable limitation was the relatively low quantitative sample size. While statistically, the case‐level sample size (*n* = 158 cases, 53 in the family interview intervention group) is sufficiently large to avoid the inflated type 1 errors sometimes associated with GEE small sample sizes (*n* < 40), it did preclude further stratification by race or socioeconomic status [27]. To address the small sample size, authors supplemented the case‐level data with item‐level data and analysis for the main outcomes, stressors identified and recommendations generated, to 166 and 840, respectively. Finally, quantitative evaluation study data related to the implementation FIMR outcomes provide suggestive rather than definitive evidence, limited by both small sample (*n* = 15) and single site.

### 4.3 Implications

#### 4.3.1 Mortality Family Interviews as a Mechanism for Achieving Health Equity

Amid growing demands for equitable healthcare quality across the globe [3], there has been progress identifying strategies for achieving this [28, 29]. At the institutional level, chief among them is centreing community priorities and diverse patient voices in the design of clinical environments and encounters. Within the United States, the Institute for Healthcare Improvement's National Plan specifically calls for patient and family interviews to understand contributors and solutions to harmful medical service events [30, 31]. In other high‐income countries, Maternal Perinatal Death Surveillance and Response (MPDSR) programmes are growing in popularity and power, and incorporate family involvement at multiple stages of the mortality review process [3, 32]. When offered the opportunity, families want to be involved in the reviews, even so far as [32] participating in the review itself [11, 33]. MPDSR programmes include the actual providers involved in the care rather than discipline representatives, which, when appropriately facilitated, can give both the provider and the family answers and closure, in addition to identifying areas for improvement in the care pathway [7, 34, 35].

#### 4.3.2 Build Upon Existing Structures to Implement Mortality Review Recommendations

Yet, administrators and policymakers alike cite the practical challenges that health systems face implementing these edicts [36, 37]. Within US FIMRs, a recommended two‐tiered structure is designed to bridge this gap, where FIMR community action teams (consisting of health system administrators and community leaders) prioritize and implement the recommendations produced by CRTs. Currently, however, only 58% of local FIMR teams report having a community action team and CRT coordinators are already stretched, with only half of their job typically allocated to FIMR duties [12]. Furthermore, while the goal of documenting both family interview details and implemented recommendations is clear from National Center for Fatality Review and Prevention guidance documents and data elements within the NFR‐CRS system, the current capacity for such action and its tracking is severely limited (https://ncfrp.org/wp-content/uploads/DataDictionary_CRS_v6-0.pdf) [5].

Integrating family interview methodology, narratives and recommendations into existing healthcare quality improvement efforts is one way to embed these strategies. A good example is UK's Perinatal Review Tool, completed for each of the 4311 perinatal UK deaths in 2023, which grades every stage in the care pathway, identifying 9922 improvement action plans that year alone [9]. While even this sophisticated system does not yet track care quality improvements made as a result of these action plans, they have initiated ‘strength’ assessments (borrowing from Root Cause Analysis Tools) [38] and provided an extensive list of quality improvement examples on their website for teams to reference (https://www.npeu.ox.ac.uk/pmrt/quality-improvement-ideas).

Study results that there are more gaps identified and recommendations made regarding clinical care and social determinants of health when an FIMR family interview is present aligns with the large body of evidence that these very issues are at the root of health disparities among Black and impoverished populations [23, 24, 39]. Tightening ties between healthcare institutions and community‐based FIMR, with its family interviews and health equity focus, could help drive equity improvements.

Beyond health systems, study findings confirm that translating mortality review recommendations and associated family interview narratives into policy‐change and culture‐shifts is a heavy lift. One avenue is to merge recommendations across multiple mortality reviews, such as FIMR, child death and maternal mortality, to strengthen existing policy pathways and elevate policies that are informed by family voice [40]. Furthermore, utilize existing funding for programmes that are poised to serve these functions. Within the United States, for instance, Title V Maternal Child Health Services programmes, combining federal and state resources, are already positioned to fulfil many translational functions: providing training and support for conducting family interviews, coordinating efforts across the different types of mortality reviews, analysing statewide mortality review recommendations, promoting state and federal legislation informed by this analysis and disseminating reports to policymakers, providers and residents [41]. Finally, secure needed family interview resources [12], through such legislation as the US Preventing Maternal Deaths Act (https://www.congress.gov/bill/118th-congress/house-bill/3838), which could mandate and fund programmes such as FIMR and MDPSR, prioritizing family interviews and requiring state departments of health to develop and disseminate prevention plans based upon FIMR recommendations [40, 42].

## 5 Conclusion

Study findings demonstrate that across methods and sources, the presence of FIMR family interviews identifies more medical service gaps and produces more recommendations, particularly regarding families' socioeconomic challenges, medical miscommunication and healthcare service improvements. Resourcing FIMRs to remove current family interview barriers, and integrating family interviews into policymaking at local, state and federal levels could centre community voice in institutional health equity efforts and policymaking at state and federal levels. Future research is needed to explore the effectiveness of various family engagement methods, corroborate study outcomes across different settings and populations and begin tracking the implementation of related recommendations as well as their subsequent impact.

## Author Contributions

**Catherine Kothari:** conceptualization, methodology, data curation, formal analysis, funding acquisition, project administration, visualization, investigation, writing – review and editing, writing – original draft. **Fernando Ospina:** methodology, data curation, investigation, formal analysis, supervision, writing – review and editing. **Nia Evans:** methodology, data curation, formal analysis, investigation, project administration, writing – review and editing. **Cynthia Bane:** data curation, writing – review and editing, methodology. **Joi Presberry Dixon:** data curation, writing – review and editing, methodology. **Vaishali Patil:** methodology, data curation, writing – review and editing. **Ruth Butters:** methodology, data curation, project administration, writing – review and editing. **Rosemary Fournier:** conceptualization, validation, funding acquisition. **Susanna C. Joy:** conceptualization, validation. **Brenda O'Rourke:** conceptualization, validation, funding acquisition. **Josephine Woods:** conceptualization, validation. **Debra Lenz:** conceptualization, validation, funding acquisition. **Aaron L. Davies:** conceptualization, validation.

## Ethics Statement

Western Michigan University Homer Stryker MD School of Medicine Institutional Review Board deemed the quantitative portion of this study (WMed IRB# 2023‐1077) as nonhuman research (per the definition of human subjects as defined by the Common Rule and FDA), and the qualitative portion of this study (WMed IRB# 2023‐1078) as meeting criteria for exempt status as described in 45 CFR Part 46.104 (d) Category 2(ii) limited review.

## Consent

Families participating in FIMR interviews were provided a written copy and verbal explanation of the purpose of the family interview that their participation was voluntary, that they have the right to stop the interview or skip questions at any time and that their personal identifying information will be kept confidential. Families were then given a chance to ask questions and decline to be interviewed. Those verbally agreeing were then asked to sign and date the written copy of the consent. The FIMR interviewer then signed and dated the consent.

## Conflicts of Interest

The authors declare no conflicts of interest.

## Data Availability

Infant mortality rate data included in this study were derived from the following resource available in the public domain: https://www.mdch.state.mi.us/osr/chi/InDx/frame.html. Qualitative deidentified transcripts are available upon reasonable request from the corresponding author. Quantitative Recommendations' data supporting study findings are available upon request from the corresponding author; they are not publicly available due to privacy and ethical restrictions. Remaining quantitative study data are not available due to privacy, regulatory and ethical restrictions.

## Appendix 1

### 1.1

Process steps in generating Kalamazoo Action Recommendations (https://www.ncfrp.org/wp-content/uploads/Kalamazoo-FIMR-Recommendations-Report-2021.pdf).

## Appendix 2

### 2.1

See Table A2.

**Table A2:** Bivariate comparison of cases with and without Life Stressor data (row percentage).

	Has stressor data (*n* = 66) 41.8% (66)	No stressor data (*n* = 92) 58.2% (92)	*p*
**Death category^**			0.42
Infant death (< 1 year)	43% (57)	57% (75)	
Stillbirth death*	69% (18)	31% (8)	
**Infant age at death^**	(*n* = 57 infants)	(*n* = 75 infants)	
Age in weeks (mean)	5.7 weeks	8.3 weeks	0.18
**Infant death cause categ.^**	(*n* = 57 infants)	(*n* = 75 infants)	0.52
Prematurity	44% (21)	55% (26)	
SUID/sleep‐related	46% (15)	55% (18)	
Congenital anomalies	52% (14)	48% (13)	
Infection/disease	*	73% (8)	
Complications (preg/delivery)	*	60% (6)	
Homicide	*	*	
Accident (non‐sleep‐related)	*	0	
**Infant death manner^**	(*n* = 57 infants)	(*n* = 75 infants)	0.31
Natural	44% (42)	56% (53)	
Accident	44% (15)	56% (19)	
Homicide	0	*	
**Infant characteristics^**			
Birthweight, grams (mean)	1380 grams	1703 grams	.12
Gestation, weeks (mean)	30.2 weeks	30.4 weeks	0.86
**Pregnancy characteristics^**			0.10
Single gestation	44% (61)	56% (77)	
Multi gestation	25% (5)	75% (15)	
**Maternal demographics^**			
Maternal age (mean)	28.8 years	27.8 years	0.31
Maternal race category			0.69
White only	38% (29)	62% (48)	
Black (only, multi)	47% (32)	53% (36)	
Latina (only, multi)	*	*	
Middle East. (only, multi)	*	*	
Native Am. (only, multi)	*		
Asian (only, multi)	0	*	
Maternal insurance status			0.83
Medicaid	35% (30)	66% (57)	
Private insurance	49% (31)	51% (32)	
Self‐pay/other [missing]	0	* [2]	
Maternal education			0.30
< High school	29% (8)	71% (20)	
HS degree	35% (18)	65% (34)	
Some college/Assoc	53% (21)	48% (19)	
Bachelor degree	44% (10)	56% (13)	
Graduate degree	60% (6)	*	

* Data suppressed if cell count < 6 to preserve confidentiality.

## Appendix 3

### 3.1

See Table A3.

**Table A3:** Comparing the NCFRS Issue responses^a^ from the family interview group within the full 158 sample and those from the family interview group within the 66 stressors sub‐sample.

	Full sample interview group (53)	Stressor subset interview group (15)	*p*
Maternal medical	84.9% (45) (	93.4%(14)	0.67
Maternal age‐related	17.0% (9)	13.3% (2)	0.73
Family planning issue	79.2% (42)	86.7% (13)	0.52
Substance use	64.2% (34)	73.3% (11)	0.51
Prenatal care	54.7% (29)	66.7% (10)	0.41
Infant medical	75.5% (40)	80.0% (12)	0.72
Paediatric care	9.4% (5)	13.3% (2)	0.66
Environment	24.5% (13)	13.3% (2)	0.36
Injury	9.4% (5)	6.7% (1)	0.74
Social support	26.4% (14)	26.7% (4)	0.98
Partner‐related issue	60.4% (32)	53.3% (8)	0.62
Family transition	32.1% (17)	20.0% (3)	0.36
Mental health, stress	75.5% (40)	73.3% (11)	0.87
Family violence, neglect	24.6% (13)	33.3% (5)	0.49
Cultural, language	11.3% (6)	13.3% (2)	0.83
Payment	86.8% (46)	93.3% (14)	0.49
Services provided	41.5% (22)	66.7% (10)	0.08

*Note:* https://ncfrp.org/wp-content/uploads/DataDictionary_CRS_v6-0.pdf.

https://www.socscistatistics.com/tests/chisquare/default2.aspx.

a Dichotomized into yes/no by combining ‘present’ and ‘contributing’ into yes.

## Appendix 4

### 4.1

See Table A4.

**Table A4:** Bivariate comparison of reasons why no interview was obtained (row percentage).

	No interview (*n* = 105)		
	Unable to Contact (*n* = 69) % (#)	Declined (*n* = 36) % (#)	*p*
**Death category**			*0.26*
Infant death (< 1 year)	64% (62)	36% (35)	
Stillbirth death	87% (7)	13% (1)	
**Infant age at death**	(*n* = 62 infants)	(*n* = 35 infants)	
Age in weeks (mean)	9.3 weeks	3.5 weeks	*0.01*
**Infant death cause categ.**	(*n* = 62 infants)	(*n* = 35 infants)	*0.15*
Prematurity	50% (18)	50% (18)	
SUID/sleep‐related	72% (21)	28% (8)	
Congenital anomalies	83% (15)	17% (3)	
Infection/disease	60% (3)	40% (2)	
Complications (preg/delivery)	57% (4)	43% (3)	
Homicide	(0)	*	
Accident (non‐sleep‐related)	*	(0)	
**Infant death manner**	(*n* = 62 infants)	(*n* = 35 infants)	*0.20*
Natural	61% (40)	39% (26)	
Accident	73% (22)	27% (8)	
Homicide	0	*	
**Infant characteristics**			
Birthweight, grams (mean)	1741 g	1329 g	*0.11*
Gestation, weeks (mean) [missing]	32.5 weeks [3]	27.9 weeks	*0.04*
**Pregnancy characteristics**			*0.55*
Single gestation	67% (61)	33% (30)	
Multi gestation	57% (8)	43% (6)	
**Perinatal care [missing]**	**66% (55) [7]**	35% (29) **[6]**	*0.27*
Preconcept. care [missing]	62% (31) [18]	38% (19) [9]	*0.40*
Postpartum visit [missing]	64% (44) [13]	36% (24) [6]	*0.60*
Preg. planning [missing]	66% (43) [18]	34% (22) [11]	*1.0*
Bereavement ref. [missing]	69% (51) [16]	31% (23) [1]	*0.11*
**Maternal demographics**			
Maternal age (mean)	28.4 years	27.9 years	*0.46*
Maternal race category			*0.13*
White only	58% (28)	42% (20)	
Black (only, multi)	75% (37)	25% (12)	
Latina (only, multi)	50% (2)	50% (2)	
Middle East. (only, multi)	*	0	
Native Am. (only, multi)	0	*	
Asian (only, multi)	0	*	
Maternal insurance status			*0.53*
Medicaid	66% (39)	34% (20)	
Private insurance	60% (23)	40% (15)	
Self‐pay/other [missing]	83% (5) [2]	17% (1)	
Maternal education			*0.69*
< High school	75% (15)	25% (5)	
HS degree	59% (20)	41% (14)	
Some college	71% (17)	29% (7)	
Associate degree	57% (4)	43% (3)	
Bachelor degree	67% (6)	33% (3)	
Masters degree	67% (4)	33% (2)	
Doctorate/prof degree [missing]	0 [3]	0 [1]	
**Svc referrals [missing]**	[27]	[8]	
Any referrals (valid %)	66% (36)	34% (19)	*0.07*
Average # of referrals (mean)	3.2 referrals	2.4 referrals	*0.15*

*Data suppressed if cell count < 6 to preserve confidentiality.
